# Thermographical Measuring of the Skin Temperature Using Laser Needle Acupuncture in Preterm Neonates

**DOI:** 10.1155/2012/614210

**Published:** 2012-05-14

**Authors:** Wolfgang Raith, Gerhard Litscher, Iris Sapetschnig, Sebastian Bauchinger, Evelyne Ziehenberger, Wilhelm Müller, Berndt Urlesberger

**Affiliations:** ^1^Division of Neonatology, Department of Paediatrics, Medical University of Graz, Auenbruggerplatz 30, 8036 Graz, Austria; ^2^Research Group for Paediatric Traditional Chinese Medicine, TCM Research Center Graz, Medical University of Graz, Auenbruggerplatz 30, 8036 Graz, Austria; ^3^Stronach Research Unit for Complementary and Integrative Laser Medicine, Research Unit of Biomedical Engineering in Anesthesia and Intensive Care Medicine, and TCM Research Center Graz, Medical University of Graz, 8036 Graz, Austria

## Abstract

In children, laser acupuncture is used more often than needle acupuncture in Western countries, due to their aversion to needles. When applying laser acupuncture to premature babies and neonates, firstly the degree of the thermal increase to the skin has to be evaluated so as to guarantee safe application. The patients were premature neonates before their discharge from hospital. The measurements were carried out by means of a polygraphy while they were asleep shortly. The large intestine 4 acupoint (LI4, *Hegu*) was stimulated by a microlaser needle (10 mW, 685 nm) twice (5 and 10 min). Local thermographic pictures were taken with a thermal camera (Flir i5, Flir Systems Inc., Portland, USA), and the warmest point was determined and subsequently compared. The study included 10 premature neonates (7 male, 3 female). The measurements were carried out on the 33rd day of life (weight 2030 g, gestational age 36 + 3 weeks of pregnancy). In comparison to the initial temperature (32.9°C), after 5 minutes of stimulation (33.9°C) (*P* = 0.025) and also after 10 minutes of stimulation (34.0°C) (*P* = 0.01), there was found to be a significant increase in the skin temperature. The singular maximum value of 37.9°C bears a potential danger; however, compared to the local temperatures reached in transcutaneous blood gas measurements it appears not to entail any risks.

## 1. Introduction

Based on the aversion children show for needles, the practice of acupuncture in children did not play a great role in therapy in traditional Chinese medicine (TCM) in Western countries. Rather, the pillars of treatment here have always been massage (*Tuina*), diets based on the five elements, and medicinal therapy. If, however, acupuncture is used in children, it is usually limited to brief light needling, or acupressure. This is the reason why, in the past, we found very little in terms of publications on this subject compared to the great interest revealed in acupuncture treatment in adults [1, 2].

However, the last few years have shown an increased interest in the complementary medical treatment of children [3, 4]. Even though, to this day, there exist only a few randomized studies on their treatment by means of acupuncture, this method is seen as efficient and safe provided it is practiced under standard conditions by well-trained acupuncturists [5, 6]. The development of laser acupuncture has opened up new dimensions altogether in the treatment of children [7]. Still, to this day, there is only very limited literature on laser acupuncture in children, with a lack of clear recommendations [8, 9].

Even if only in individual cases, acupuncture in neonates [10] has been carried out as a therapy to combat infantile colic [11–13] applying a light needling technique using single use needles and giving mild stimulation. Laser acupuncture provides a noninvasive therapeutic approach, thus excluding the risk of infection caused by needle prick injuries [14, 15]. Nevertheless, depending on the relevant laser classification, laser acupuncture cannot be seen as not being dangerous at all since it bears a potential risk for the eyes and the surface of the skin [16]. Since it is especially the skin of premature babies and neonates that exhibits various histological and physiological peculiarities and, additionally, a normalization of the sweating function only sets in over a period of time of the first 6–8 months of life [17], exposure to laser acupuncture and, going hand in hand with it, a warming of the skin would indeed subject this very sensitive patient population to a particularly high risk.

It was the aim of the study to explore whether or not acupuncture by means of laser significantly changes the surface temperature, thus representing a potential risk of application given the circumstances. 

## 2. Materials and Methods

### 2.1. Probands

The probands were former premature babies of the Division of Neonatology at the Graz University Clinic Department of Pediatrics who all underwent tests in the sleep lab before being discharged from hospital. Their parents were informed about the examination and gave their prior written consent. The study itself was submitted to the Ethics Committee of the Medical University of Graz and approved.

### 2.2. Laser Acupuncture

The probands were comfortably placed on a Babytherm 8000 incubator (Dräger GmbH, Lübeck, Germany) in the course of the sleep lab examination. In all incidents, a time period of 10 min of waiting was respected before applying the laser needles to give the skin of the neonates a chance to stabilize temperaturewise. The laser needle used for acupuncture (Laserneedle EG GmbH, Berlin, Germany) provides continuous laser light with a wavelength of 685 nm and an output power of 10 mW per laser needle.

Then, after a waiting period of 25 min, laser needle acupuncture was performed simultaneously in both arms on the large intestine 4 (*Hegu*) point. The first stimulation carried out lasted for 5 min. After an interval of 10 min, a second stimulation was carried out in the same way, but this time lasting for 10 min.

We attempted to select an acupoint that is one of the most commonly used acupuncture points in TCM: large intestine 4 (LI4, *Hegu*). LI4 (*Hegu*) is located in the large intestine meridian in the middle of the 2nd metacarpal bone on the radial side. There are a total of 20 points on the large intestine meridian. The pathway begins on the index finger and travels along the arm, over the shoulder to end on the face just to the outside of the nose.

### 2.3. Thermography

Before laser acupuncture application, as well as 1 min, 5 min, and 10 min after, respectively, thermographic pictures of both the left and right hands were taken by means of a thermal camera (Flir i5, Flir Systems Inc., Portland, USA). Subsequently, the warmest spot was identified and reidentified and compared in the course of time.

**Figure 1:**
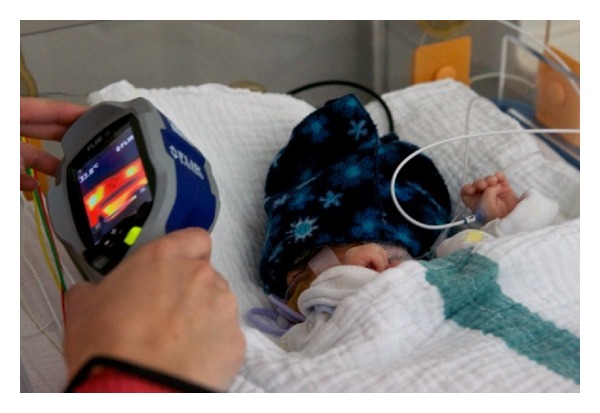
Infrared analytic measurement setup.

**Figure 2:**
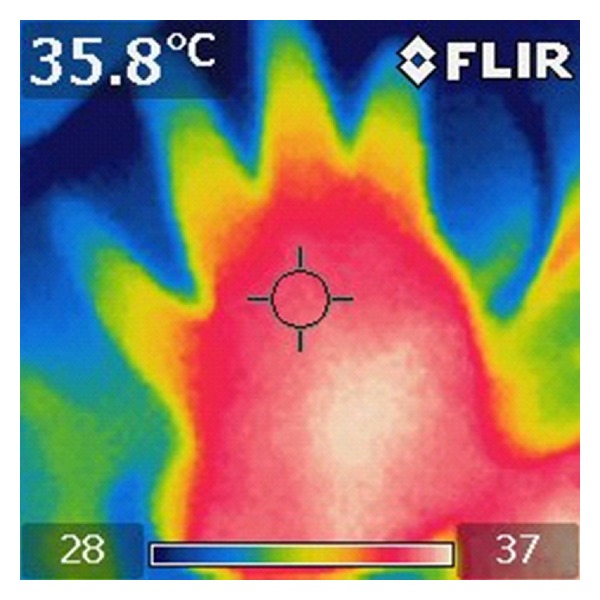
Thermogram of the hand after laser needle stimulation.

Figure 1 shows the use of the thermal camera during the examination, and Figure 2 gives an example of the pictures taken with the use of the thermal camera.

Additionally in the course of the examination, any parameters measurable in the context of polygraphy, such as the heart rate, oxygen saturation, end-expiratory CO_2_, and breathing movements including electroencephalography (EEG), were recorded and analyzed. Throughout the examination, the ambient temperature and humidity were kept constant. Atthatpoint in time of the examination, no medication modifying the blood circulation was administered.

### 2.4. Statistics

All data was taken as a mean value ± SD (standard deviation). The statistical evaluation was done using ANOVA test for repeated measurings and the Tukey test, respectively.

## 3. Results

All together 10 neonates (7 male, 3 female, gestational age (GA) 31 + 5 weeks of pregnancy, birth weight 1703 g) were included in the study. On average, the measurings were carried out on the 33rd day after birth (weight at that point in time of examination 2030 g, GA 36 + 3 weeks of pregnancy).

Table 1 gives the data of the children involved in the study. 

Altogether, 20 thermographical measurings were taken of the above-mentioned measuring points. Compared to the initial temperature of 32.9°C, the skin temperature had significantly risen to 33.9°C (*P* = 0.025) after 5 min of stimulation. Equally, a significant rise in temperature was measured again after 10 min of stimulation (34.0°C) (*P* = 0.01). The maximum measured skin temperature after stimulation was found to be 37.9°C. The parameters comeasured during the examination (heart rate, oxygen saturation, end-expiratory CO_2_, and breathing movements including EEG) showed no significant changes.

**Figure 3:**
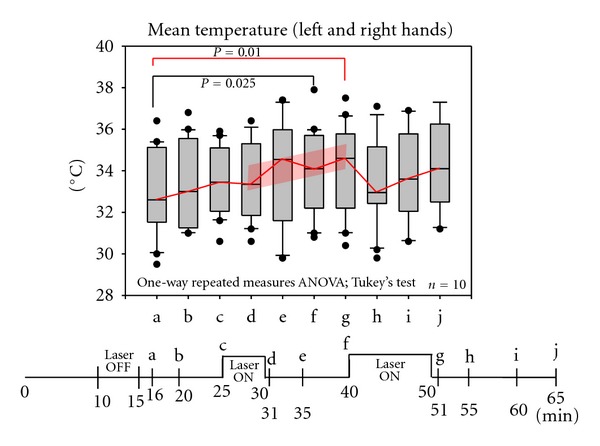
Results and time curve of the examination.

Figure 3 gives the results and time curve of the examination.

## 4. Discussion

In the context of acupuncture research, not only central but also peripheral effects, such as any changes in the blood supply of the skin due to acupuncture treatment, play a major role. Any changes in surface temperature can be made visible with the help of thermography. It was the aim of this study to obtain, for the first time, effective data on changes in surface temperature in neonates from laser acupuncture. Thermography, that is, the temperature measured by means of an infrared camera, represents a measuring procedure that has been used before in adults for acupunctural research. The wavelength range of thermal and infrared energy lies beyond the perception threshold of the human eye. Rather, the energy is found in the section of the electromagnetic range perceived by humans as warmth. In contrast to visible light, each object in this segment whose temperature lies above absolute zero radiates heat; the higher the temperature of an object, the more intense the infrared radiation it emits. Infrared cameras produce pictures of the otherwise invisible infrared or heat radiation and allow for very accurate temperature measurements.

Crucial advantages of this procedure lie in the optical and thus touch-free data collection and in the visualization of temperature distributions of a high local resolution. Moreover, the examination technique is of a passive nature, that is, no additional energy is supplied to the body. It is therefore totally harmless and fit to be applied on neonates [18].

The laser acupuncture needles (Laserneedle) [19] are glued to the skin for the acupuncture treatment, which means that pricking is not required. Also, these special needles allow for the simultaneous stimulation of individual point combinations or for the simultaneous stimulation of paired points. The stimulation of more than one acupuncture point using different laser acupunctural gadgets has so far only been possible one after the other, not simultaneously. Both its simple application and basic research up to now have well-established laser needle acupuncture in adults in acupunctural research [20–22].

The point selected for the examination under this study, LI4 (*Hegu*) can be quickly and easily found in adults and neonates alike. LI4 (*Hegu*), is considered one of the most effective acupuncture points for general pain control. Additionally, due to its analgetic effect, the point is applied in neonates suffering from infantile colic, an effect that has been confirmed by several studies obtained so far [11–13, 23].

With the present study carried out by us in 10 former premature babies, a significant rise in skin temperature both after 5 min and after 10 min of laser acupuncture treatment using laser needle 10 mW/685 nm was measured in the point LI4 (*Hegu*) compared to the initial temperature of 32.9°C.

Especially in premature babies and neonates whose skin is altogether thin and shows physiological and histological peculiarities, it is important to note that there is a potential danger of damage to the skin. On the one hand, the skin of neonates in the first few months of life contains the so-called fetal collagen (type III) and less elastin in proportion to the skin of adults. On the other hand, due to a lack of dermal papilla, there is a reduced dermoepidermal interaction of epidermis and dermis. While the *stratum basale* immediately after birth runs almost parallel to the surface, increasingly in the course of the first few months dermal papilla is formed, a process which finally has the effect that a smooth interaction of both skin layers is achieved [24]. On top of that, the body function of thermal sweating is not fully mature in neonates, that is, the induction threshold to sweating in them is higher than in adults. In fact, the limit value of the induction threshold is dependent on the gestation age. With premature babies, we find anhidrosis in the first few days of life, with the sweating function only normalising in the course of the first 6–8 months of life [25]. It is these histological and physiological differences between premature babies and neonates on the one hand, and adults on the other hand, that explain the increased sensitivity of the skin of neonates showing, for example, in more redness of the skin and a higher likeliness for hematoma following needle acupuncture [26].

On average, a rise in the local temperature by 1 degree Celsius was found to occur after 5–10 min of laser acupuncture using laser needle 10 mW/685 nm. The highest measured skin temperature after stimulation amounted to 37.9°C. However, compared to the local temperatures reached in transcutaneous blood gas measuring [27] still used in neonatal intensive care units in the monitoring of premature babies and neonates [28, 29], the warming of the skin after 5 and 10 min, respectively, of laser needle acupuncture using laser needle (10 mW/685 nm) seems not to represent any risks.

Several studies in the field of both manual needle acupuncture and electrically stimulated acupuncture have revealed local and general warming effects as an indicator of a reduced sympathetic activity [30, 31]. However, the question as to whether or not changes in the surface temperature in connection with the application of laser acupuncture in premature babies and neonates are to be understood as changes in the sympathetic activity and therefore as an effect directly resulting from acupuncture could not be answered by this study. In the parameters additionally measured in the course of the examinations under this study (heart rate, oxygen saturation, end-expiratory CO_2_, breathing movements including EEG) no significant changes were found.

## 5. Conclusion

By way of conclusion it can be said that a significantly raised skin temperature was measured both after 5 and after 10 min of application of local laser needle acupuncture. On average, this rise in local temperature amounted to 1 degree Celsius. Although only found once, the maximum temperature measured of 37.9°C revealed the potential danger of a local warming of the skin. Nevertheless, compared to the local temperatures reached in transcutaneous blood gas measuring [27], laser needle acupuncture applied by way of the procedure described above appears not to bear any risks.

## Figures and Tables

**Table 1:** Demographic data of the 10 participants of the study.

Number of patients	10
(male/female)	(7/3)
Mean GA	31 weeks + 5 days
Mean birth weight	1703 g (SD = 513.4)
Mean arterial PH from umbilical cord sampling	7.28 (SD = 0.06)
Mean APGAR 1	6.7 (SD = 1.19)
Mean APGAR 5	8.3 (SD = 1.1)
mean APGAR 10	9 (SD = 0.63)
Mean corrected GA, at the time of investigation	36 weeks + 3 days
Mean postgestational age at the time of investigation	33 days (SD = 22)
Mean weight at the time of investigation	2030 g (SD = 250.8)

GA: gestational age; SD: standard deviation of the mean; APGAR: Apgar-Score.

## References

[1] Cao J, Su X (1900). *Essentials of Traditional Chinese Pediatrics*.

[2] Hou JL, Geng C (1995). *Treatment of Pediatric Diseases in Traditional Chinese Medicine*.

[3] Kemper KJ, Sarah R, Silver-Highfield E, Xiarhos E, Barnes L, Berde C (2000). On pins and needles? Pediatric pain patients’ experience with acupuncture. *Pediatrics*.

[4] Tsao JC, Meldrum M, Bursch B, Jacob MC, Kim SC, Zeltzer LK (2005). Treatment expectations for CAM interventions in pediatric chronic pain patients and their parents. *Evidence-based Complementary and Alternative Medicine*.

[5] Adams D, Cheng F, Jou H, Aung S, Yasui Y, Vohra S (2011). The safety of pediatric acupuncture: a systematic review. *Pediatrics*.

[6] Gentry KR, McGinn KL, Kundu A, Lynn AM Acupuncture therapy for infants: a preliminary report on reasons for consultation, feasibility, and tolerability.

[7] Pothmann R (2008). What can be achieved by using acupuncture and related techniques as well as diatetics and chinese herbal and drug therapy in paediatrics?. *German Journal of Acupuncture & Related Techniques*.

[8] Raith W, Schmölzer GM, Resch B, Seewann M, Mueller W, Urlesberger B (2008). Laser acupuncture as a possible treatment for an agitated Infant — a preterm after 28 weeks of gestation. *German Journal of Acupuncture & Related Techniques*.

[9] Loo M (2002). *Pediatric Acupuncture*.

[10] Thiel MT, Stockert K (2011). Acupuncture and Neonatology. *Journal of Chinese Medicine*.

[11] Landgren K, Kvorning N, Hallström I (2010). Acupuncture reduces crying in infants with infantile colic: a randomised, controlled, blind clinical study. *Acupuncture in Medicine*.

[12] Reinthal M, Andersson S, Gustafsson M (2008). Effects of minimal acupuncture in children with infantile colic—a prospective, quasi-randomised single blind controlled trial. *Acupuncture in Medicine*.

[13] Skjeie H, Skonnord T, Fetveit A, Brekke M (2011). A pilot study of ST36 acupuncture for infantile colic. *Acupuncture in Medicine*.

[14] Ernst E, White A (1997). Life-threatening adverse reactions after acupuncture? a systematic review. *Pain*.

[15] Peuker ET, White A, Ernst E, Pera F, Filler TJ (1999). Traumatic complications of acupuncture: therapists need to know human anatomy. *Archives of Family Medicine*.

[16] Pötinen P, Pothman R (2005). *Laser in Acupuncture*.

[17] Schachner LA, Hansen RC (2011). *Pediatric Dermatology: Expert Consult (Cohen, Pediatric Dermatology)*.

[18] Frankenberger RT, Bussmann O, Nahm W, Konecny E, Gortner L (1998). Measuring lateral skin temperature profile of premature infants in incubators with thermography. *Biomedical Technology*.

[19] Litscher G, Schikora D (2005). *Laserneedle-Acupuncture. Science and Practice*.

[20] Litscher G (2009). Ten years evidence based high-tech acupuncture—a short review of centrally measured effects. *Evidence-Based Complementary and Alternative Medicine*.

[21] Litscher G (2009). Ten years evidence based high-tech acupuncture—a short review of peripherally measured effects. *Evidence-based Complementary and Alternative Medicine*.

[22] Litscher G (2006). Bioengineering assessment of acupuncture, part 1: thermography. *Critical Reviews in Biomedical Engineering*.

[23] Reinthal M, Lund I, Ullman D, Lundeberg T (2011). Gastrointestinal symptoms of infantile colic and their change after light needling of acupuncture:a case series study of 913 infants. *Chinese Medicine*.

[24] Hoeger PH, Enzmann CC (2002). Skin physiology of the neonate and young infant: a prospective study of functional skin parameters during early infancy. *Pediatric Dermatology*.

[25] Fluhr JW, Darlenski R, Taieb A (2010). Functional skin adaptation in infancy—almost complete but not fully competent. *Experimental Dermatology*.

[26] Jindal V, Ge A, Mansky JP (2008). Safety and efficacy of acupuncture in children: a review of the evidence. *Journal of Pediatric Hematology/Oncology*.

[27] Golden MS (1981). Skin craters—a complication of transcutaneous oxygen monitoring. *Pediatrics*.

[28] Poets CF, Martin R, Stocks J, Sly PD, Tepper RS, Morgan WJ (1996). Noninvasive determination of blood gases. *Infant Respiratory Function Testing*.

[29] Rüdiger M, Töpfer K, Hammer H, Schmalisch G, Wauer RR (2005). A survey of transcutaneous blood gas monitoring among European neonatal intensive care units. *BMC Pediatrics*.

[30] Ernst M, Lee MH (1985). Sympathetic vasomotor changes induced by manual and electrical acupuncture of the Hoku point visualized by thermography. *Pain*.

[31] Ernst M, Lee MH (1986). Sympathetic effects of manual and electrical acupuncture of the Tsusanli knee point: comparison with the Hoku hand point sympathetic effects. *Experimental Neurology*.

